# Effects of fasting on the expression pattern of FGFs in different skeletal muscle fibre types and sexes in mice

**DOI:** 10.1186/s13293-020-00287-7

**Published:** 2020-03-10

**Authors:** Wei-hua Jia, Nuo-qi Wang, Lin Yin, Xi Chen, Bi-yu Hou, Jin-hua Wang, Gui-fen Qiang, Chi Bun Chan, Xiu-ying Yang, Guan-hua Du

**Affiliations:** 1 State Key Laboratory of Bioactive Substance and Function of Natural Medicines and Beijing Key Laboratory of Drug Target and Screening Research, Institute of Materia Medica of Peking Union Medical College, Beijing, 100050 China; 2 State Key Laboratory of Pharmaceutical Biotechnology, School of Biological Sciences, The University of Hong Kong, Hong Kong, China

**Keywords:** Fasting, Fibroblast growth factor, Skeletal muscle, Fibre type, Sex, Mice

## Abstract

Fibroblast growth factors (FGFs) belong to a large family comprising 22 FGF polypeptides that are widely expressed in tissues. Most of the FGFs can be secreted and involved in the regulation of skeletal muscle function and structure. However, the role of fasting on FGF expression pattern in skeletal muscles remains unknown. In this study, we combined bioinformatics analysis and in vivo studies to explore the effect of 24-h fasting on the expression of *Fgfs* in slow-twitch soleus and fast-twitch tibialis anterior (TA) muscle from male and female C57BL/6 mice. We found that fasting significantly affected the expression of many *Fgfs* in mouse skeletal muscle. Furthermore, skeletal muscle fibre type and sex also influenced *Fgf* expression and response to fasting. We observed that in both male and female mice fasting reduced *Fgf6* and *Fgf11* in the TA muscle rather than the soleus. Moreover, fasting reduced *Fgf8* expression in the soleus and TA muscles in female mice rather than in male mice. Fasting also increased *Fgf21* expression in female soleus muscle and female and male plasma. Fasting reduced *Fgf2* and *Fgf18* expression levels without fibre-type and sex-dependent effects in mice. We further found that fasting decreased the expression of an FGF activation marker gene—*Flrt2* in the TA muscle but not in the soleus muscle in both male and female mice. This study revealed the expression profile of *Fgfs* in different skeletal muscle fibre types and different sexes and provides clues to the interaction between the skeletal muscle and other organs, which deserves future investigations.

## Introduction

Fibroblast growth factors (FGFs) are a large family of polypeptide growth factors that are widely expressed in organisms ranging from nematodes to humans [1]. FGFs regulate complex biological functions in vivo and in vitro, including embryogenesis, mitogenesis, cellular migration and differentiation, angiogenesis, wound healing, skeleton growth, energy homeostasis, metabolism, and adipogenesis [2].

In human, the FGF family contains 22 FGF proteins, four FGF receptors (FGFR1-4) [2], and one receptor (FGFR5) lacking an intracellular kinase domain [3]. There is no human *FGF15* gene; the gene orthologous to mouse *FGF15* is *FGF19* [4]. FGFs are classified into three major groups [5]: Canonical FGFs (FGF1-FGF10, FGF16-FGF18, FGF20 and FGF22) can be secreted from cells. Intracrine FGFs (FGF11-FGF14) are not secreted but participate in intracellular processes independent of FGFRs [6]. Hormone-like FGFs (FGF15/19, FGF21 and FGF23) have systemic effects [1, 7].

Studies show that fasting has several healthy benefits [8], such as increasing longevity, alleviating obesity and other non-infectious diseases [9], and anti-tumour [10]. However, fasting can also lead to muscle wasting and cause life-threatening problems [11]. These effects are closely related to the physiological response of the skeletal muscle to fasting. The skeletal muscle is not only a motor organ, but also a metabolic organ, which stores most of the body’s energy and regulates systemic metabolism. During fasting or energy deficits, the skeletal muscles would adjust energy demands and preserve energy homeostasis [12]. Ours and other studies found that growth factors coordinate metabolic reprogramming in muscle during fasting [13].

FGFs have long been implicated in skeletal muscle structure and function [14]. Several FGFs have been proved to function as myokines, such as FGF2 [15] and FgF21 [15, 16]. A myokine is a protein or peptide that it is derived and secreted from the skeletal muscle and performs biological functions in an endocrine or paracrine manner [17]. FGFs were initially associated with myogenesis [18]. FGFs, FGFRs and their co-receptor (heparin or heparan sulfate) are expressed in a time- and space-dependent manner during all stages of skeletal development [19]. FGF15/19 was found to ameliorate skeletal muscle atrophy [20]. Studies reported that Fgf21 expressed by muscle controls muscle mass [16] and increases insulin sensitivity [21].

The skeletal muscles of mammals are composed of multiple muscle fibre types, including oxidative slow-twitch (type I), mixed oxidative-glycolytic fast-twitch (type IIa) and glycolytic fast-twitch (type IIb) myofibres, which differ in their metabolic properties [22]. The soleus muscle has a higher proportion of type I muscle fibres than that of many other muscles [23], and the tibialis anterior (TA) muscle has significantly more type IIb fibres [24]. Studies report that fasting influences FGF expression in the skeletal muscle. It was observed that fasting and metabolic disorders induced FGF21 release from muscles [16], whereas the muscle *Fgf6* mRNA levels were not significantly affected by animal feeding status [25]. In addition, biological sexes have different responses to muscle health, largely due to the significant variety of general muscle fibre type, satellite cell activation and proliferation, anabolic and catabolic factors, and hormonal interactions between males and females [26]. Our previous study found that *Fgf15* expression is higher in the skeletal muscle of female mice than that of male mice. Fasting reduces *Fgf15* expression in female muscles but had no effect on male muscles [27].

However, the mechanisms by which fasting influences the expression pattern of skeletal muscle FGFs is still unknown. In this study, we investigated the expression of all the 22 *Fgfs* in slow-twitch soleus and fast-twitch tibialis anterior (TA) muscle, to explore the influence of muscle fibre types and sexes on *Fgf* expression during fasting in female and male mice.

## Materials and methods

### Animals

This study and all the procedures using animals were approved by the Animal Care and Use Committee of the Institute of Materia Medica, Chinese Academy of Medical Sciences. Female and male C57BL/6 mice (20–22 g), obtained from the Institute of Laboratory Animal Science, Chinese Academy of Medical Sciences (Beijing, China), were used in this study. Mice were housed in environmentally controlled conditions with a 12-h light/dark cycle at a temperature of 22 ± 3 °C and humidity of 55 ± 5%. Mice were provided free access to food and water for 7 days before the experiment.

### Animal fasting procedure

After being housed for 7 days, female and male C57BL/6 mice were randomly allocated either to a fed group (Fed) or a fasted group (Fast) with six mice in each group. The fasted mice were placed in new cages without providing any food in the cage at 9 A.M. After fasting for 24 h [16, 28, 29], the Fed and Fast mice were anaesthetised with isoflurane, blood samples were collected via the abdominal vein and the indicated skeletal muscles from different anatomical positions were quickly removed and frozen in liquid nitrogen for RNA extraction and further experiments.

### Quantitative reverse transcription PCR

Total RNA was isolated from ~ 50 mg muscles or a whole piece of the soleus muscle by homogenisation in Trizol Isolation Reagent (Invitrogen, USA), and then purified with Direct-zol RNA Kits (cat. no. R2052, ZYMO search, USA) as previously described [27, 30]. Reverse transcription was performed using 1 μg of total RNA with a reverse transcription reaction mix that contained Superscript III reverse transcriptase (Invitrogen, USA) and Oligo-dT17 as primers. Specific mRNA content was determined using AceQ qPCR SYBR Green Master Mix (cat. no. Q111-03, Vazyme, Biotech) on a CFX-96 Real-time PCR System (Bio-Rad, USA) with gene-specific primer pairs (Table 1). The total reaction volume was 10 μl. The results were quantified after normalisation with TATA-box binding protein (*Tbp*) [31, 32].

**Table 1:** Primers used for real-time PCR

Pairs	Genes	Primer sequence
1	Mouse *Fgf1* 5′	CCCTGACCGAGAGGTTCAAC
	Mouse *Fgf1* 3′	GTCCCTTGTCCCATCCACG
2	Mouse *Fgf2* 5′	GCGACCCACACGTCAAACTA
	Mouse *Fgf2* 3′	TCCCTTGATAGACACAACTCCTC
3	Mouse *Fgf3* 5′	TACAACGCAGAGTGTGAGTTTG
	Mouse *Fgf3* 3′	CACCGACACGTACCAAGGTC
4	Mouse *Fgf4* 5′	TACCCCGGTATGTTCATGGC
	Mouse *Fgf4* 3′	TTACCTTCATGGTAGGCGACA
5	Mouse *Fgf5* 5′	AAGTAGCGCGACGTTTTCTTC
	Mouse *Fgf5* 3′	CTGGAAACTGCTATGTTCCGAG
6	Mouse *Fgf6* 5′	CAGGCTCTCGTCTTCTTAGGC
	Mouse *Fgf6* 3′	AATAGCCGCTTTCCCAATTCA
7	Mouse *Fgf7* 5′	CTCTACAGATCATGCTTCCACC
	Mouse *Fgf7* 3′	ACAGAACAGTCTTCTCACCCT
8	Mouse *Fgf8* 5′	AGAGCCTGGTGACGGATCA
	Mouse *Fgf8* 3′	CTTCCAAAAGTATCGGTCTCCAC
9	Mouse *Fgf9* 5′	ATGGCTCCCTTAGGTGAAGTT
	Mouse *Fgf9* 3′	TCCGCCTGAGAATCCCCTTT
10	Mouse *Fgf10* 5′	TTTGGTGTCTTCGTTCCCTGT
	Mouse *Fgf10* 3′	TAGCTCCGCACATGCCTTC
11	Mouse *Fgf11* 5′	TAGCCTGATCCGACAGAAGC
	Mouse *Fgf11* 3′	GGCAGAACAGTTTGGTGACG
12	Mouse *Fgf12* 5′	CAGGCCGTGCATGGTTTCTA
	Mouse *Fgf12* 3′	TCGTGTAGTGATGGTTCTCTGT
13	Mouse *Fgf13* 5′	CTCATCCGGCAAAAGAGACAA
	Mouse *Fgf13* 3′	TTGGAGCCAAAGAGTTTGACC
14	Mouse *Fgf14* 5′	CCCCAGCTCAAGGGCATAG
	Mouse *Fgf14* 3′	TGATGGGTAGAGGTAACCTTCTC
15	Mouse *Fgf15* 5′	ATGGCGAGAAAGTGGAACGG
	Mouse *Fgf15* 3′	CTGACACAGACTGGGATTGCT
16	Mouse *Fgf16* 5′	GTGTTTTCCGGGAACAGTTTGA
	Mouse *Fgf16* 3′	GGTGAGCCGTCTTTATTCAGG
17	Mouse *Fgf17* 5′	GGCAGAGAGCGAGAAGTACAT
	Mouse *Fgf17* 3′	CGGTGAACACGCAGTCTTTG
18	Mouse *Fgf18* 5′	CCTGCACTTGCCTGTGTTTAC
	Mouse *Fgf18* 3′	TGCTTCCGACTCACATCATCT
19	Mouse *Fgf20* 5′	GGTGGGGTCGCACTTCTTG
	Mouse *Fgf20* 3′	GATACCGAAGAGACTGTGATCCT
20	Mouse *Fgf21* 5′	CTGCTGGGGGTCTACCAAG
	Mouse *Fgf21* 3′	CTGCGCCTACCACTGTTCC
21	Mouse *Fgf22* 5′	CCAGGACAGTATAGTGGAGATCC
	Mouse *Fgf22* 3′	AGTAGACCCGCGACCCATAG
22	Mouse *Fgf23* 5′	ATGCTAGGGACCTGCCTTAGA
	Mouse *Fgf23* 3′	AGCCAAGCAATGGGGAAGTG
23	Mouse *Flrt2* 5′	ATGGGCCTACAGACTACAAAGT
	Mouse *Flrt2* 3′	CAGCGGCATACACTAGGGC
24	Mouse *Flrt3* 5′	CCTCATCGGGACTAAAATTGGG
	Mouse *Flrt3* 3′	GCAAGTTCTTCAAATCGGAAGGA
25	Mouse *Tbp* 5′	ACCCTTCACCAATGACTCCTATG
	Mouse *Tbp* 3′	ATGATGACTGCAGCAAATCGC

### Measurement of FGFs in mice plasma

Plasma concentrations of FGF15, FGF21, FGF23, and FLRT2 were quantified using the ELISA kits (cat.no. CSB-EL522052MO, CUSABIO; cat.no.ab212160, Abcam; cat.no.ab213863, Abcam; cat.no. JL38212, Shanghai Jianglai Biotech) according to the manufacturer’s instructions, respectively.

### GEO data acquisition and FGF expression analysis

We first performed human tissue-specific *FGF* expression analysis in non-diseased tissues using Genotype-Tissue Expression (GTEx) expression dataset (https://gtexportal.org/home/multiGeneQueryPage), which is a comprehensive public resource to study tissue-specific gene expression and regulation. For each gene, expression values were normalised across samples using an inverse normal transform. Next, we explored *Fgf* expression in the skeletal muscle in mice using the Gene Expression Omnibus (GEO) database. The GEO is a public functional genomics data repository that provides a multimodal data repository and retrieval system for microarray and next-generation sequencing gene expression profiles. This study queried and downloaded the relevant studies from the GEO website (The Gene Expression Omnibus, https://www.ncbi.nlm.nih.gov/gds/) [33]. We identified a suitable dataset comparing expression in the skeletal muscles of fed and fasting mice, which is an RNA sequencing dataset (accession number GSE107787). In this dataset study, male C57BL/6 mice at 8 weeks of age were randomly divided into an ad libitum fed group (*n* = 18) or a 24-h fasted group (*n* = 18). For complete experimental details, please refer to a previous publication [29].

### Statistical analysis

Differences between the groups were tested using unpaired two-tailed Student’s *t* test or one-way ANOVA. Data were analysed using GraphPad Prism (GraphPad Software, USA). Data are presented as means ± S.E.M. The significance level was set at *P* ≤ 0.05.

## Results

### Bioinformatic analysis found FGFs expressed differently and could be influenced diversely by fasting

**Fig. 1:**
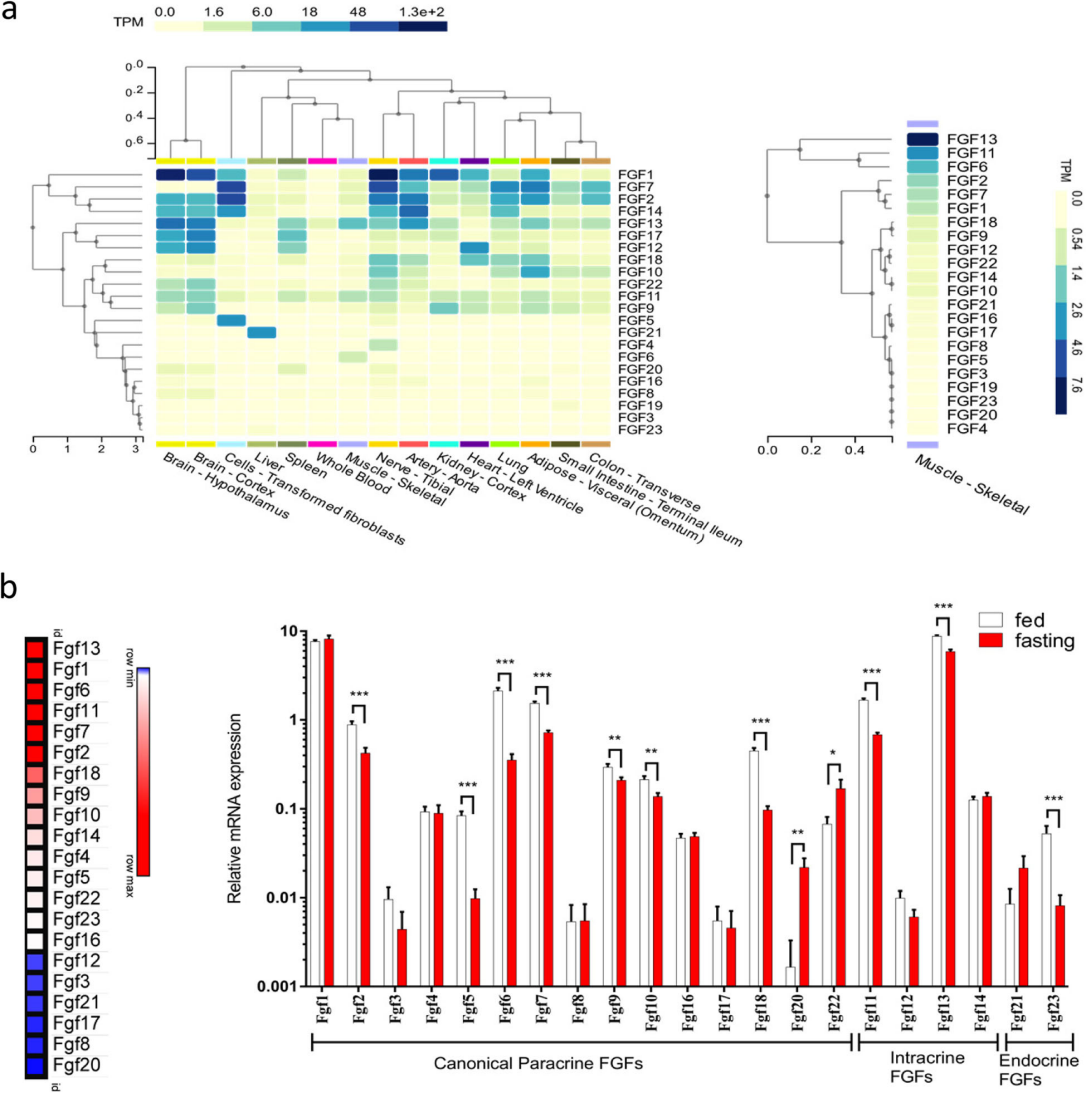
Bioinformatics analysis demonstrates that fasting influences *FGF* expression in skeletal muscles. **a** Human *FGF* expression levels in skeletal muscles compared with those in other tissues. Left: The expression of *FGFs* in various tissues. Right: Heatmap of *FGF* expression in skeletal muscles. **b** Expression of *Fgfs* in muscle from 24-h fasted mice. Left: Heatmap of basal expression of *Fgfs* in skeletal muscles. Right: Expression of *Fgfs* in gastrocnemius muscle. Data represent means ± S.E.M, *n* = 18; biological replicates. *P < 0.05, **P < 0.01 and *** *P* < 0.001 by one-way ANOVA followed by unpaired two-tailed Student’s *t* test

We compared expression levels of human *FGFs* in the skeletal muscle with that in other tissues. Bioinformatic results from GTEx dataset show that *FGFs* were expressed divergently in different tissues. Different *FGFs* demonstrated distinctive expression in the human skeletal muscle, of which *FGF13*, *FGF11*, *FGF6*, *FGF2* and *FGF7* had the top five expression levels (Fig. 1a). Next, fasting-induced expressions of *Fgfs* in the skeletal muscle of male mice were analysed using a dataset from NCBI Gene Expression Omnibus (GEO). In this dataset, mice were fasted for 24 h and the gastrocnemius skeletal muscles were taken for RNA sequencing experiment (accession numbers GSE107787) [29]. According to the analysis of the dataset results, the top five *Fgf* genes with the highest expression levels in mice gastrocnemius were *Fgf13*, *Fgf1*, *Fgf6*, *Fgf11* and *Fgf7*. Meanwhile, in the case of canonical FGFs, fasting induced *Fgf20* and *Fgf22* expression by 13.2- and 2.5-fold and reduced *Fgf2*, *Fgf5*, *Fgf6*, *Fgf7*, *Fgf9*, *Fgf10* and *Fgf18* expression by 2.1-, 8.5-, 6.0-, 2.1-, 1.4-, 1.6- and 4.6-fold, respectively. Regarding intracellular FGFs, fasting reduced *Fgf11*, *Fgf13* expression by 2.5- and 1.5-fold. The expression of an endocrine FGF—Fgf23—was reduced by 6.4-fold (Fig. 1b). However, data on *Fgf15* was not obtained from the RNA sequencing results.

### Differential expression of* Fgf*s in male and female mice according to fibre type of the skeletal muscle

**Fig. 2:**
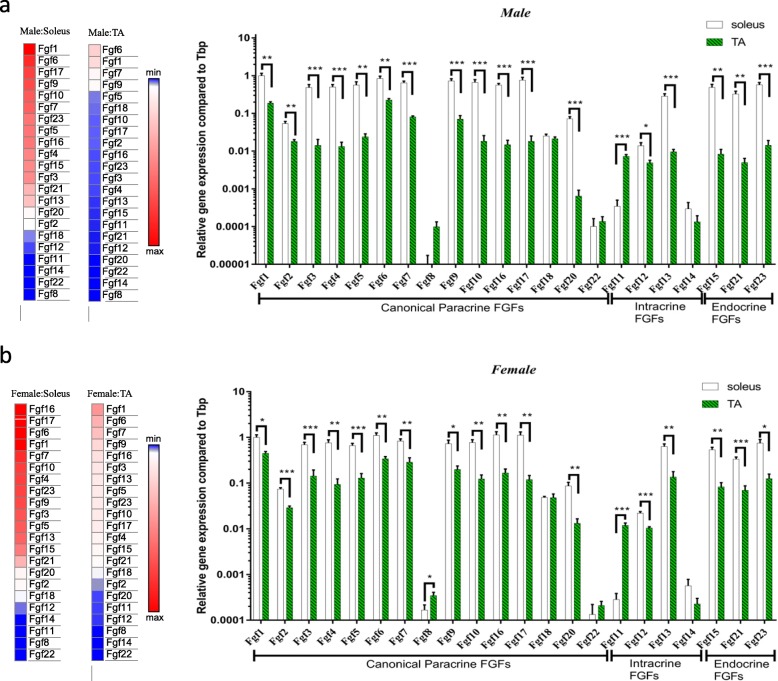
*Fgf* expression levels in different muscle fibre types of male and female C57BL/6 mice. **a** Basal *Fgf* expression level in male mice. Left: Heatmap of basal *Fgf* expression in the TA and soleus muscles. Right: Comparison of *Fgf* expression levels between the soleus and TA muscles. **b** Basal *Fgf* expression level in female mice. Left: Heatmap of basal *Fgf* expression in the TA and soleus muscles. Right: Comparison of *Fgf* expression levels between the soleus and TA muscles. Data represent means ± S.E.M, *n* = 6; biological replicates. *P < 0.05, **P < 0.01 and ***P < 0.001 by one-way ANOVA followed by unpaired two-tailed Student’s *t* test

Our previous study showed that slow-twitch soleus and fast-twitch TA have different myokine expressions [27]. In this study, we found that the muscle fibre types also influenced expressions of *Fgfs*. The results showed that in male C57BL/6 mice, expressions of canonical *Fgf1*–*Fgf7*, *Fgf9*, *Fgf10*, *Fgf16*, *Fgf17* and *Fgf20* in the soleus muscle were 5.3-, 3.0-, 34.4-, 37.8-, 23.4-, 3.7-, 8.0-, 10.3-, 36.3-, 38.0-, 41.9- and 112.4-fold higher compared with those in the TA muscle, respectively. Intracellular *Fgf12* and *Fgf13* expressed in the soleus muscle were 2.8- and 29.8-fold higher than those in the TA muscle, while *Fgf11* in the TA muscle was 21.2-fold higher than that in the soleus muscle. Expressions of endocrine *Fgf15*, *Fgf21* and *Fgf23* in the soleus muscle were 59.0-, 65.1- and 39.7-fold higher than those of the TA muscle (Fig. 2a).

In female C57BL/6 mice, canonical *Fgf1*-*Fgf7*, *Fgf9*, *Fgf10*, *Fgf16*, *Fgf17* and *Fgf20* had 2.2-, 2.6-, 4.9-, 8.2-, 5.2-, 3.3-, 2.9-, 3.7-, 6.3-, 6.8-, 9.4- and 6.6-fold higher expression in the soleus than those in the TA muscle, respectively, which is consistent with male mice. *Fgf8* and *Fgf11* in the TA muscle were 2.1- and 43.5-fold higher than those in the soleus muscle. Intracellular *Fgf12* was expressed in the soleus muscle as 2.1-fold as much as that in the TA muscle, while *Fgf11* in the TA muscle was 43.5-fold higher than that in the soleus muscle. Expressions of endocrine *Fgf15*, *Fgf21* and *Fgf23* in the soleus muscle were 6.5-, 4.8- and 6.0-fold higher than those in the TA muscle, respectively (Fig. 2b).

### *Fgf* expression in the TA and soleus muscles during fasting in male C57BL/6 mice

**Fig. 3:**
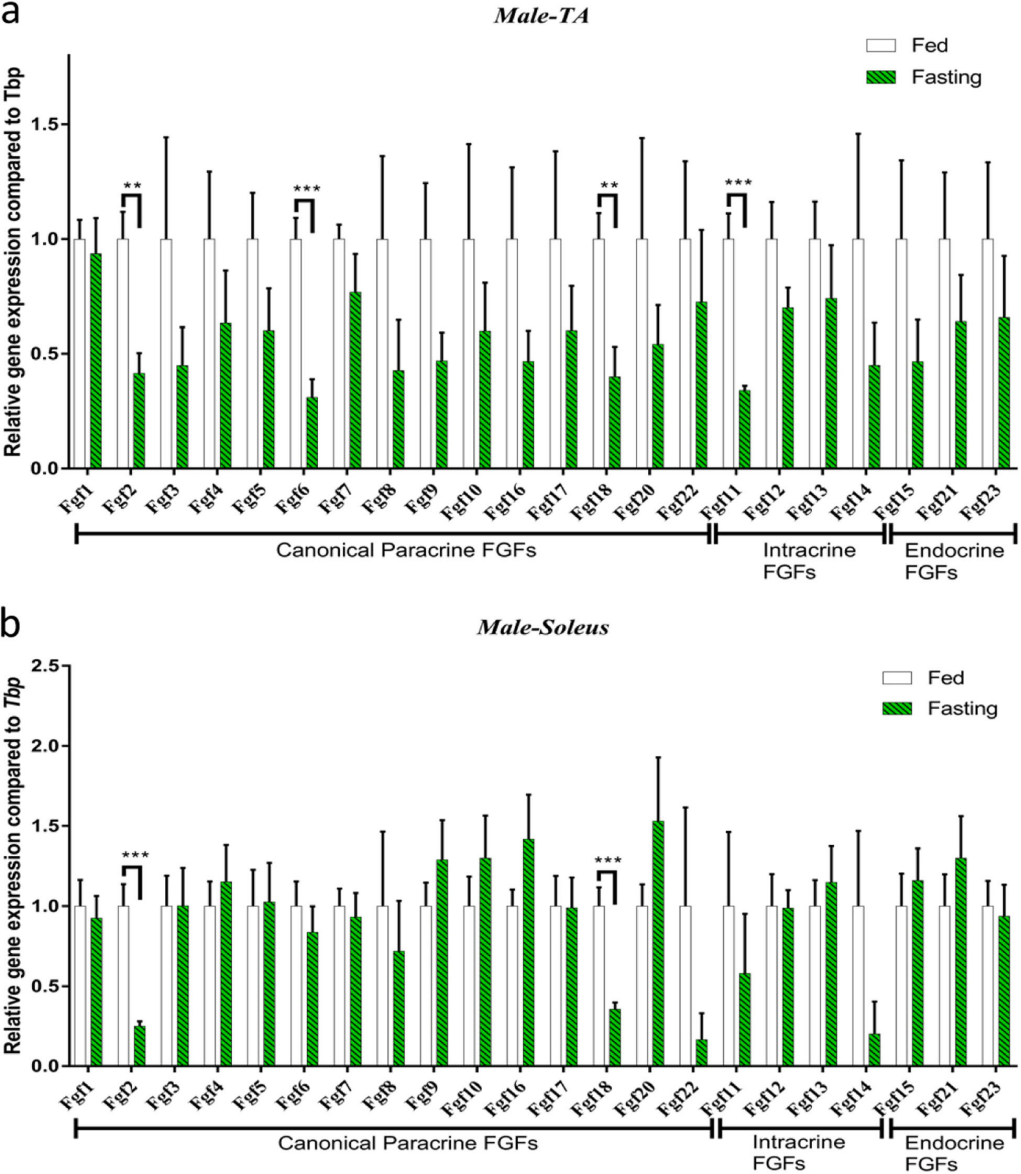
Fasting-stimulated *Fgf* expression pattern changes were different in the TA and soleus muscles in male C57BL/6 mice. **a***Fgf* expression in the TA muscle. **b***Fgf* expression in the soleus muscle. Data represent means ± S.E.M, *n* = 6; biological replicates. *P < 0.05, **P < 0.01 and *** *P* < 0.001 by one-way ANOVA followed by unpaired two-tailed Student’s *t* test

In this study, we analysed all expression levels of *Fgfs* in the TA and soleus muscles of fasting male C57BL/6 mice. Our study showed that fasting influenced the *Fgf* expression of the TA and soleus. In fast-twitch TA muscle, fasting reduced canonical *Fgf2*, *Fgf6* and *Fgf18* expression by 2.4-, 3.2- and 2.5-fold, respectively. Intracellular *Fgf11* was reduced by 2.9-fold during fasting (Fig. 3a). On the contrary, in slow-twitch soleus muscle, 24-h fasting induced the expression of *Fgf2* and *Fgf18* by 4.0- and 2.8-fold, respectively (Fig. 3b).

### *Fgf* expression in the TA and soleus muscles during fasting in female C57BL/6 mice

**Fig. 4:**
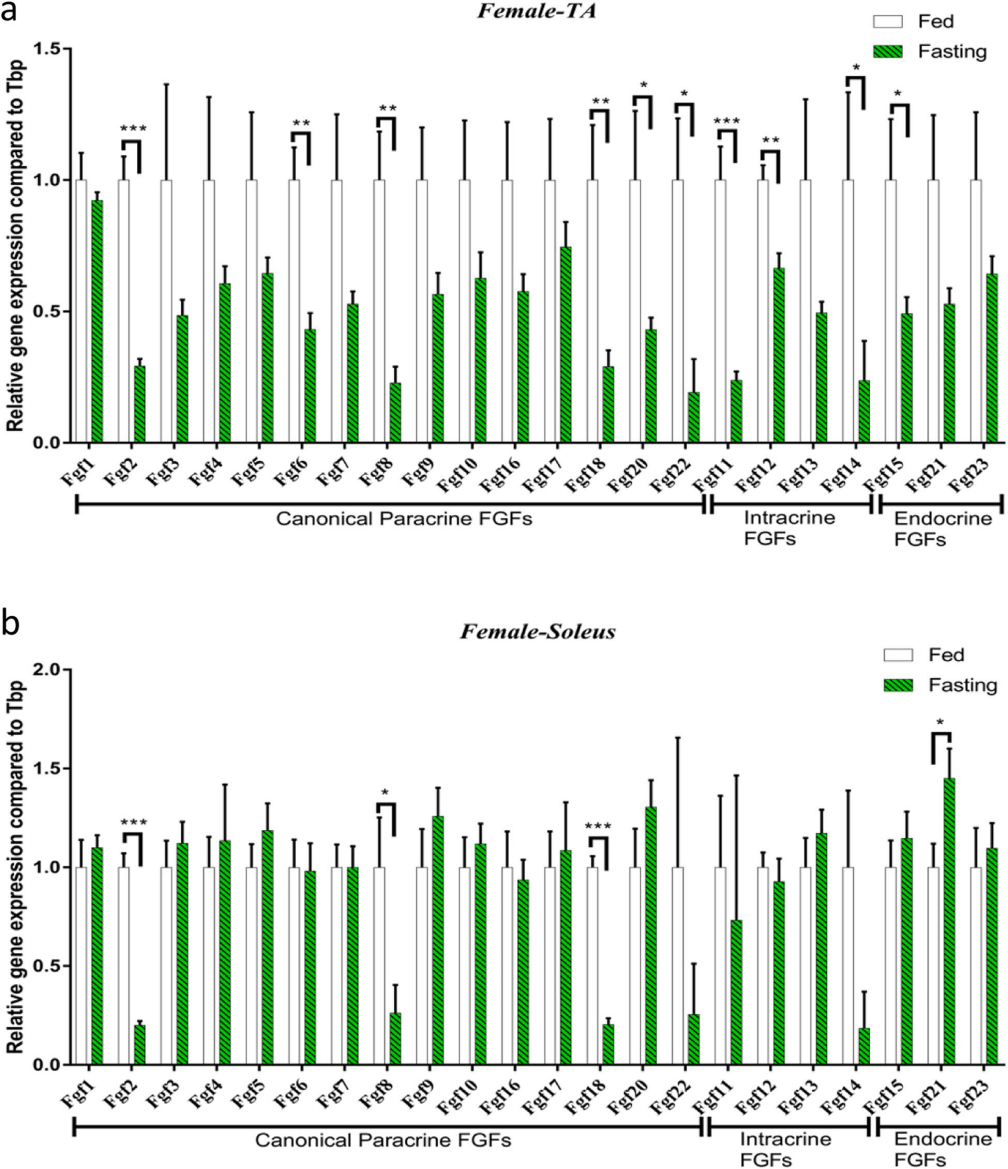
Fasting-stimulated *Fgf* expression pattern changes were different in the TA and soleus muscles in female C57BL/6 mice. **a***Fgf* expression in the TA muscle. **b***Fgf* expression in the soleus muscle. Data represent means ± S.E.M, *n* = 6; biological replicates. *P < 0.05, **P < 0.01 and ***P < 0.001 by one-way ANOVA followed by unpaired two-tailed Student’s *t* test

In this study, the effects of fasting on the pattern of *Fgf* expression were different in female C57BL/6 mice compared with those in male mice. In fast-twitch TA muscle, as for canonical FGFs, 24-h fasting reduced *Fgf2*, *Fgf6*, *Fgf8*, *Fgf18* and *Fgf20* by 3.4-, 2.3-, 4.4-, 3.4- and 2.3-fold, respectively. Intracellular *Fgf11*, *Fgf12* and *Fgf14* were reduced by 4.2-, 1.5- and 4.2-fold, respectively. Endocrine *Fgf15* and *Fgf23* were reduced by 2.0- and 1.6-fold (Fig. 4a). In slow-twitch soleus muscle, 24-h fasting reduced the expression of *Fgf2*, *Fgf8* and *Fgf18* by 5.0-, 3.8- and 4.9-fold, respectively, while endocrine *Fgf21* was 1.9-fold higher than that of fed soleus muscle (Fig. 4b).

### Differential expression of *Fgf*s in the soleus and TA muscles according to the sex of mice

**Fig. 5:**
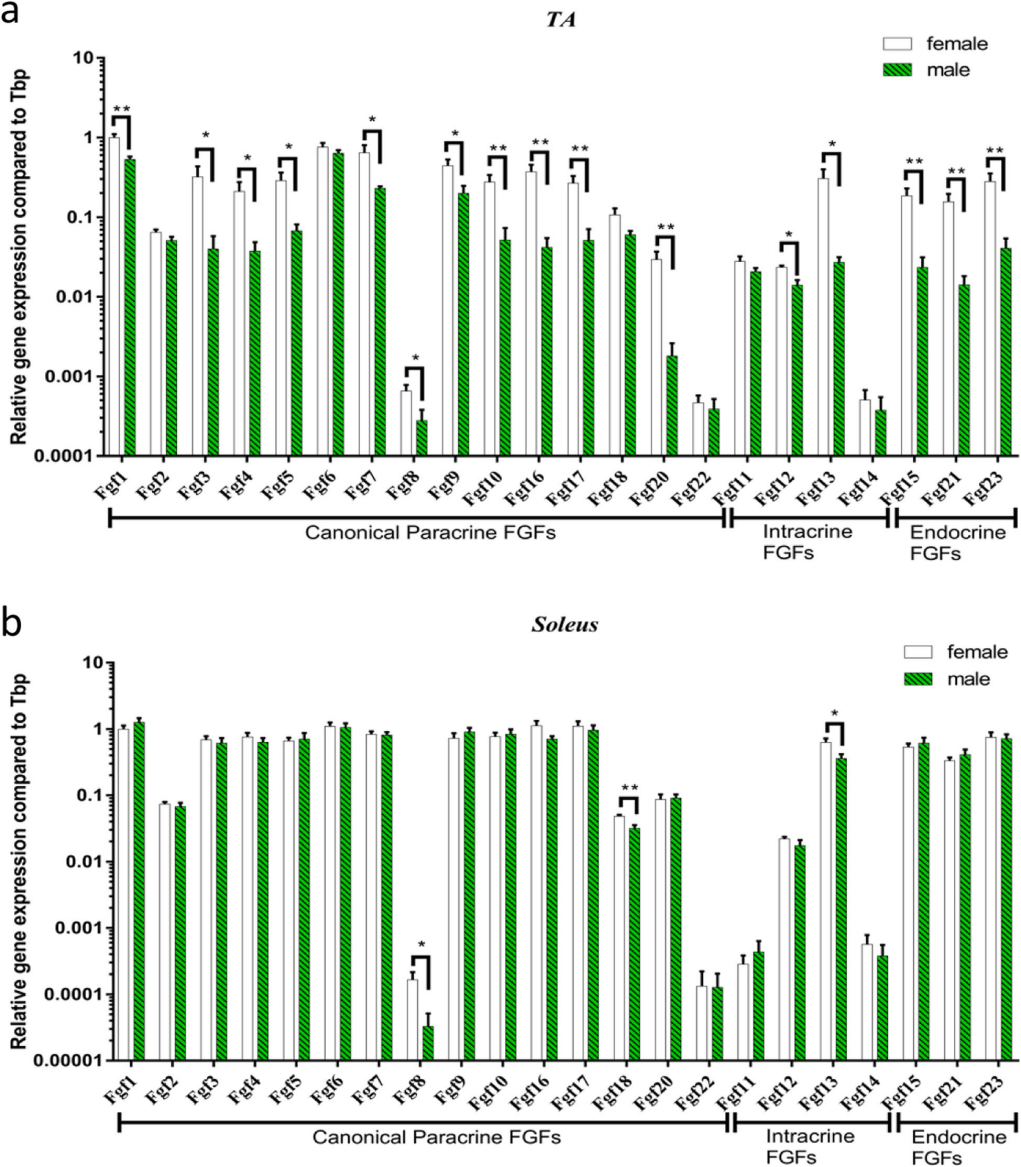
Expression of *Fgfs* in skeletal muscles were divergent in male and female C57BL/6 mice. **a** Comparison of *Fgf* expression levels in the TA muscle. **b** Comparison of *Fgf* expression levels in the soleus muscle. Data represent means ± S.E.M, *n* = 6; biological replicates. *P < 0.05, **P < 0.01 and ***P < 0.001 by one-way ANOVA followed by unpaired two-tailed Student’s *t* test

The expression of *Fgfs* in the soleus muscle was similar in male and female mice (Fig. 5a), while they were dramatically different in the TA muscle (Fig. 5b). Moreover, most of the *Fgf* expressions were higher in female TA muscle compared with those of male TA muscle, including canonical *Fgf1*, *Fgf3*–*Fgf5*, *Fgf7*–*Fgf10*, *Fgf16*, *Fgf17* and *Fgf20*; intracellular *Fgf12* and *Fgf13*; and endocrine *Fgf15, Fgf21* and *Fgf23*.

### Differential expression of *Flrt*s in the soleus and TA muscles of male and female mice

**Fig. 6:**
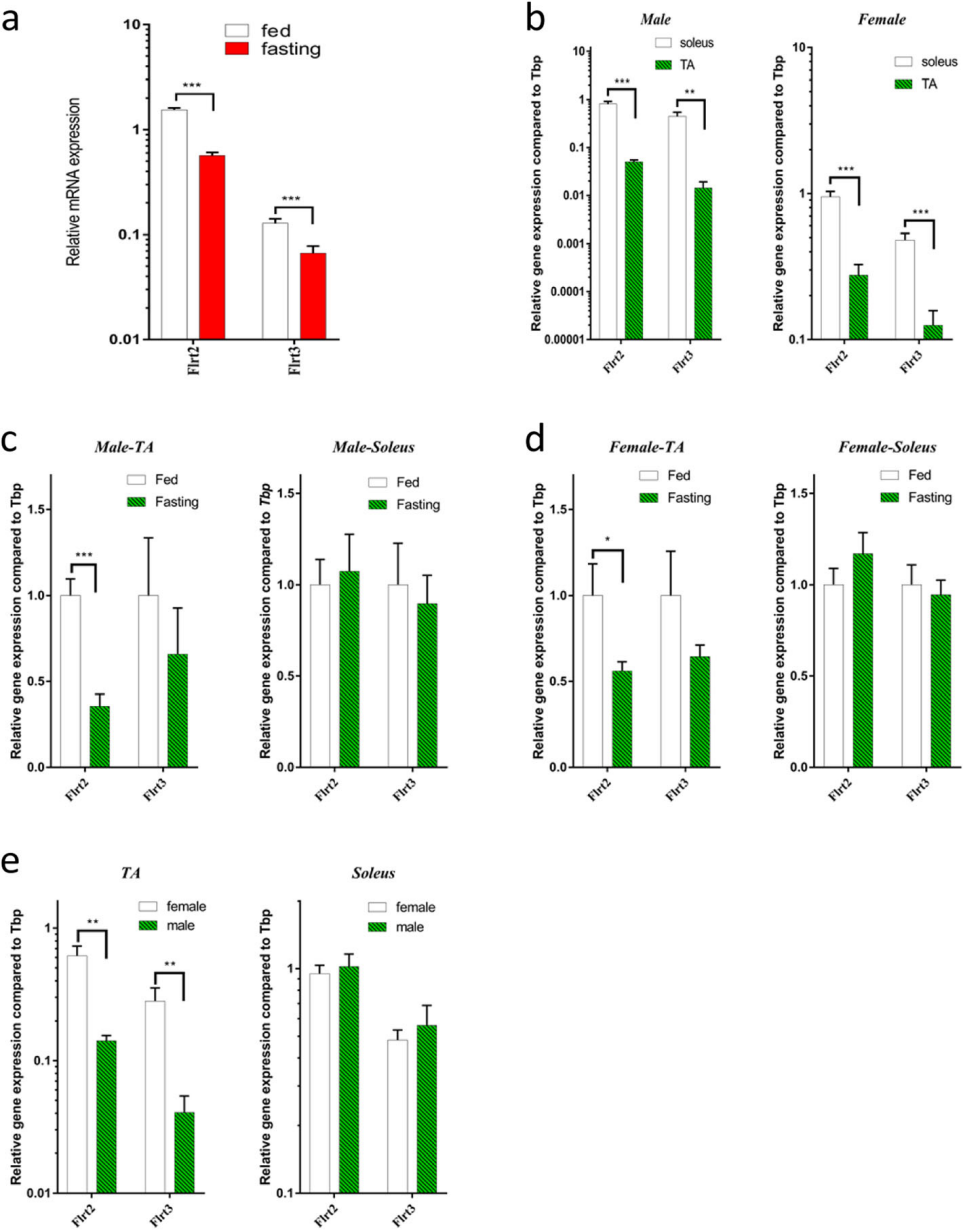
Fasting-stimulated *Flrt* expression pattern in C57BL/6 mice. **a** Expression of *Flrts* in gastrocnemius muscle from 24-h fasted mice. **b** Basal *Flrt* expression levels in different muscle fibre types of male (light) and female (right) C57BL/6 mice. **c** Fasting-stimulated *Flrt* expression in the TA muscle of male (light) and female (right) C57BL/6 mice. **d** Fasting-stimulated *Flrt* expression in the soleus muscle of male (light) and female (right) C57BL/6 mice. **e** Fasting-stimulated *Flrt* expression in the TA (light) and soleus (right) muscles of male and female C57BL/6 mice. Data represent means ± S.E.M, *n* = 6; biological replicates. *P < 0.05, **P < 0.01 and ***P < 0.001 by one-way ANOVA with unpaired two-tailed Student’s *t* test

Fibronectin-leucine-rich transmembrane proteins (FLRTs) are markers of FGF activity [34, 35]. Fasting reduced *Flrt2* and *Flrt3* expression by 2.8- and 1.7-fold in mice gastrocnemius skeletal muscle (Fig. 6a). In male C57BL/6 mice, *Flrt2* and *Flrt3* in the soleus muscle were 16.1- and 30.8-fold higher than those of the TA muscle, and so is in female mice, *Flrt2* and *Flrt3* in the soleus muscle were 3.4- and 3.8-fold higher than those of the TA muscle (Fig. 6b). Furthermore, in male C57BL/6 mice, fasting reduced *Flrt2* expression in the TA muscle, while fasting did not affect *Flrt2* expression in the soleus muscle (Fig. 6c). Female C57BL/6 mice showed similar changes, fasting also reduced *Flrt2* expression by 1.8-fold in the TA muscle and had no effect on the soleus muscle (Fig. 6d). In addition, in the TA muscle, basal *Flrt2* and *Flrt3* express more in female mice than in male C57BL/6 mice, while there is no difference in the soleus muscle (Fig. 6e).

### Endocrine FGF15, FGF21 and FGF23 levels in mice plasma

**Fig. 7:**
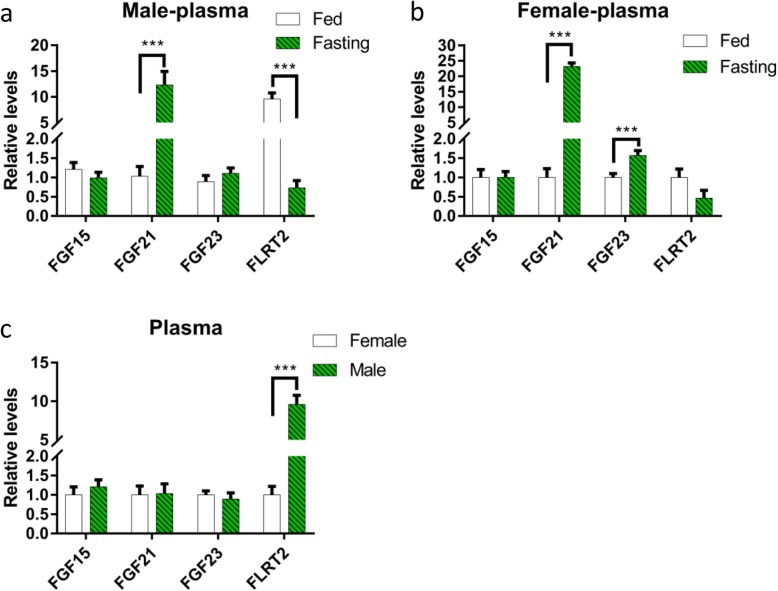
Expression of endocrine FGFs and FLRT2 in plasma. **a** Comparison of FGF15, FGF21, FGF23 and FLRT2 expression levels in response to fasting in male mice plasma. **b** Comparison of FGF15, FGF21, FGF23 and FLRT2 expression levels in response to fasting in female mice plasma. **c** Comparison of FGF15, FGF21, FGF23 and FLRT2 expression levels in female and male mice plasma. Data represent means ± S.E.M, *n* = 6; biological replicates. *P < 0.05, **P < 0.01 and ***P < 0.001 by one-way ANOVA with unpaired two-tailed Student’s *t* test

Endocrine FGFs have systemic effects via blood circulation, and plasma endocrine FGF concentration may alter according to condition changes or diseases. In several situations, they can act as biomarkers for some diseases [6]. Fasting significantly increased FGF21 circulating level in both male and female mice by 11.9- and 23.2-fold, respectively. FGF23 increased by 1.6-fold in female mice only. As for FLRTs, we measured FLRT2 and found it decreased by 13.1-fold in respond to fasting in male mice, but slightly decreased in female mice with no significant difference (Fig. 7).

## Discussion

Fasting and fed conditions divergently influence skeletal muscle structure and metabolism [36]. In this study, we found that fasting influenced expressions of *Fgfs* in mouse skeletal muscle. Furthermore, sex and skeletal muscle fibre type also influenced *Fgf* expression and the response to fasting.

The 15 canonical FGFs (FGF1–FGF10, FGF16–FGF18, FGF20 and FGF22) act predominantly in an autocrine and paracrine fashion and bind to FGFRs in a complex with heparan sulfate proteoglycans (HSPGs) [37]. In this study, we found that fasting reduced *Fgf2* and *Fgf18* expression levels in both fibre types and sexes in mice. This result is consistent with a previous study that revealed that in fish, fgf2 mRNA level increased by 2.5-fold at both 4 and 12 days after refeeding compared to that in fasting conditions [38]. FGF2 released from skeletal muscle cells plays important roles in myogenesis and muscle development [39]. FGF2 contributes to muscle compensatory growth induced by refeeding [38]. Extracts from slow-twitch muscles contained higher levels of FGF2 than those from fast-twitch muscles [40]. This is in line with our results, that in both male and female mice, the expression levels of all *Fgfs* were higher or comparable in slow-twitch soleus muscle compared with those in fast-twitch TA muscle. What’s more, fasting reduced *Fgf6* expression in the TA muscle rather than the soleus in both the male and female. Fasting reduced *Fgf8* expression in female rather than male mice in both the TA and soleus muscles. Thus far, effects of fasting on the expression levels of other canonical *Fgfs*, except *Fgf2*, in the skeletal muscle have not been reported.

The intracellular FGFs (FGF11–FGF14) are non-secretory forms that bind and modulate voltage-gated sodium channels (VGSCs) [41]. *Fgf13* is highly expressed in muscle and inhibits C2C12 cell proliferation and differentiation [42]. In our study, we found that fasting decreased *Fgf13* expression in gastrocnemius skeletal muscle. In addition, it is of interest that *Fgf11* is expressed more in the TA than in the soleus, which differs from any other Fgfs. And fasting reduced *Fgf11* expression in both the male and female TA muscle rather than the soleus. Fasting reduced *Fgf12* and *Fgf14* expression levels in female soleus muscle other than TA. However, there have no reports related to the effects of fasting on intracellular FGF expression so far.

The three endocrine FGFs (FGF15/19, FGF21 and FGF23) act as hormones and lack affinity for HSPG binding, which enables their diffusion from the site of production into the circulation. Endocrine FGFs are expressed in various tissues and organs. They play roles in cell growth, differentiation, bile acid, glucose and lipid metabolism, as well as in the control of vitamin D and phosphate levels, thereby maintaining whole-body homeostasis [43, 44]. Secretory forms of FGFs directly and indirectly control the differentiation of fast- and slow-twitch muscle lineages, respectively [45]. The three endocrine FGF mRNA expressions are higher in the soleus than those in the TA in both sexes, and as for TA, they are higher in female than those in male. Oestrogen can increase hepatic production of FGF21, suggesting that sex influences gene expression [46]. In this study, we found that fasting reduced *Fgf15* mRNA level in female TA muscle, while had no notably influence on the protein level in plasma. FGF15/19 induced skeletal muscle hypertrophy and blocked muscle atrophy [20]. FGF15/19 is mainly secreted from the small intestine in response to feed [47]. FGF21 has been reported negatively to regulate muscle mass and contribute towards skeletal muscle atrophy [16]. In this study, we found that fasting increased *Fgf21* mRNA expression level in female soleus muscle and increased FGF21 level in plasma in both female and male mice. Emerging evidence has shown that fasting increases hepatic *Fgf21* mRNA expression and plasma FGF21 level in mice [47]. FGF21 plays a role in fasting-induced muscle atrophy and weakness. However, a report suggested that fasting significantly decreased plasma FGF21 in obese subjects [48]. Circadian regulation has a stronger impact on plasma FGF21 than that of fasting within a 72-h period [49]. We also found that fasting decreased *Fgf23* mRNA expression in gastrocnemius skeletal muscle in male mice, but increased FGF23 protein expression in female mice plasma. FGF23 is a bone-derived factor [50] and plays a role in metabolic diseases [51]. FGF23 induces cellular senescence in human skeletal muscle mesenchymal stem cells [52]. A study indicated that the expression of FGF23 is higher in females than in males [53], while we found no significant difference in the protein expression between two sexes.

Regarding the FGFR activation marker gene, we found that *Flrts* express more in the soleus than those in the TA, and fasting decreased the expression of *Flrts* in the TA muscle in two sexes, while in the soleus, the expression of *Flrts* was not significantly affected. As for FLRT2 in plasma, fasting reduced its concentration in male mice. What’s more, *Flrt* expression was sex-differentiated. There were more *Flrts* in female than in male mice TA muscle, while no sexual differences in the soleus muscle. However, in plasma, there is more FLRT2 protein expression in male mice. Thus, our study shows that the effect of fasting on FGF activity is closely related to the fibre type of skeletal muscle.

However, the lack of the data on FGF protein expressions in muscle is a limitation of this study. Our future work will focus on the related mechanisms and implications of individual FGFs on the function and structure of the skeletal muscle.

## Perspectives and significance

This study provides a new insight into the effects of fasting on FGF expression in skeletal muscles. In addition, our study uncovers the expression profiles of FGFs in different muscle fibre types and sexes. The knowledge of the biological characteristics of FGF mRNA expression is critical for the research on skeletal muscle. This may also help to identify new biomarker in skeletal muscles and novel therapeutics targeting on fasting related health benefits.

## Data Availability

The datasets used and/or analysed during the current study are available from the corresponding author on reasonable request.

## Abbreviations

FGFs: Fibroblast growth factors
FLRT: Fibronectin-leucine-rich transmembrane
GEO: Gene Expression Omnibus
GTEx: Genotype-Tissue Expression
HSPGs: Heparan sulfate proteoglycans
TA: Tibialis anterior
VGSCs: Voltage-gated sodium channels
